# Encapsulation Techniques to Enhance Astaxanthin Utilization as Functional Feed Ingredient

**DOI:** 10.3390/md23040143

**Published:** 2025-03-26

**Authors:** Matteo Vitale, Joaquin Gomez-Estaca, Janete Chung, Seong-Chea Chua, Daniela Maria Pampanin

**Affiliations:** 1 Department of Chemistry, Bioscience, and Environmental Engineering, University of Stavanger, 4021 Stavanger, Norway; daniela.m.pampanin@uis.no; 2 Skretting Aquaculture Innovation, 4016 Stavanger, Norway; janete.chung@skretting.com (J.C.); seong-chea.chua@skretting.com (S.-C.C.); 3 Instituto de Ciencia y Tecnologia de Alimentos y Nutricion (ICTAN-CSIC), 28040 Madrid, Spain

**Keywords:** astaxanthin, aquaculture, encapsulation, *in vitro* testing, fish cell lines

## Abstract

Herein, the effectiveness of astaxanthin (AX) as functional feed ingredient was assessed by enhancing its stability and bioavailability using encapsulation methods. Spray-drying and liposome entrapment were applied to a natural AX source from shrimp by-products, along with two commercially synthetic alternatives. Encapsulated AX formulations were evaluated for their physico-chemical properties, thermal stability, and *in vitro* performance using RTL-W1, a rainbow trout (*Oncorhynchus mykiss*) liver-derived cell line. Both techniques achieved high encapsulation efficiency (73–89%) and provided remarkable protection to AX during thermal treatments, maintaining its stability at 80 °C for up to 2 h and at 100 °C for 30 min. Nevertheless, neither encapsulation methods significantly mitigated water absorption over time. Additionally, morphological characterization revealed spray-dried microcapsules with typical round, partially collapsed particles with a broad size distribution, while liposomes further stabilized into dry powders by spray-drying showed structural rearrangements and an increase in size upon rehydration, although maintaining a uniform and stable distribution. *In vitro* testing revealed enhanced RTL-W1 cell viability and reduced reactive oxygen species (ROS) production when encapsulation was employed. Overall, these findings demonstrate the potential of the selected encapsulation techniques to optimize the stability, bioavailability, and functionality of AX, providing valuable insights to improve its utilization as a functional ingredient in fish feed formulations.

## 1. Introduction

Astaxanthin (AX, 3′3-dihydroxy-β-carotene-4′4-dione), a xanthophyll carotenoid, is a widespread pigment included in salmonid, crustacean, and other farmed fish feeds, as they lack the ability to synthesize carotenoids de novo and must acquire them from the diet [1]. In aquaculture, AX is commonly employed for flesh pigmentation, providing the desirable reddish-orange colour in salmonids, shrimps, and lobsters, shaping consumer perception and market acceptance [2,3,4]. Beyond its use and many beneficial properties, AX is particularly renowned for its strong antioxidant activity, which surpasses that of β-carotene and even α-tocopherol [5,6], protecting fish from oxidative stress, enhancing immune responses, and improving growth performance and overall fish health [7]. However, despite its commercial significance, the effective application of AX in aquaculture remains challenged by its low water solubility and susceptibility to oxidation and environmental conditions (temperature, light, oxygen, pH changes), which result in low bioavailability and significant losses during feed processing [8,9].

Commercial production of AX predominantly relies on synthetic sources (>95%), primarily obtained through chemical synthesis due to its cost-effectiveness and availability [10,11]. The rising consumer demand for natural and sustainable alternatives has driven interest in AX derived from biological sources, such as algae, yeast, fungi, bacteria, crustaceans, and fish [12]. The main biological source of AX is the microalga *Haematococcus pluvialis* [13], recognized as the major producer of AX (about 4% of dry weight) and widely employed at an industrial scale [14]. Additionally, AX production has also been reported in *Rhodotorula toruloides* (syn. *Rhodosporidium toruloides*) [15] and *Xanthophyllomyces dendrorhous* (syn. *Phaffia rhodozyma*) [16] yeast, as well as in *Paracoccus carotinifaciens* bacteria [17], and in by-products of the fish industry. Crustacean by-products, particularly from *Litopenaeus vannamei* shrimp processing, have emerged as a promising and sustainable alternative, supporting circular economy principles, where materials are continuously reused rather than discarded, supporting more sustainable production practices [18]. Shrimp by-products (cephalothorax, cuticles, pleopods, tails), which can account for up to 50–60% of body weight, are rich in polyunsaturated fatty acids (PUFAs), α-tocopherol, cholesterol, and carotenoids, including AX [19]. Extracting these lipids provides an effective way to valorise this waste into a valuable functional ingredient [18,19,20,21]. Nevertheless, despite their growing relevance, natural sources remain limited by higher production costs, extraction complexities, and sensitivity to environmental degradation [10], necessitating innovative strategies to enhance scalability and cost-effectiveness, while improving stability and functionality in industrial applications.

Encapsulation, a process in which active compounds are enclosed within a material forming capsules, has been widely employed as a promising technological approach to address these challenges, playing a crucial role in AX stabilization, protecting it from degradation, improving bioavailability, and enhancing its retention during digestion and feed processing [22,23]. Several encapsulation techniques have been explored, including spray-drying, emulsification, liposome entrapment, and complex coacervation, among others [24]. Among these, spray-drying, a method used to convert liquid formulations into dry powders by rapidly drying atomizing droplets, is widely employed in the food and feed industries due to its cost-effectiveness and scalability [25]. Alternatively, an innovative approach involves the use of liposomes to encapsulate bioactive compounds like AX, providing unique advantages such as controlled release, enhanced bioavailability, and biocompatibility due to their phospholipid bilayer structure [26,27]. However, the large-scale application of such methods in aquafeeds remains challenged by economic feasibility, process efficiency, and scalability.

Although previous studies have investigated the potential of encapsulation methods for AX [13,28,29,30,31], only a few have systematically compared different techniques, particularly in terms of their impact on physico-chemical stability and biological functionality in aquaculture applications. Moreover, despite the increasing interest in *in vitro* approaches as predictive tools for evaluating functional ingredients, there is a lack of information on their use in assessing the cellular-level benefits of encapsulation during early-stage evaluations, where they play a crucial role in enhancing sustainability of aquaculture practices by reducing reliance on *in vivo* trials and minimizing environmental impact.

The present study aimed to evaluate two different encapsulation techniques—spray-drying and liposome entrapment—applied to AX derived from shrimp by-products, along with two commercially synthetic alternatives. The encapsulated formulations were evaluated in terms of encapsulation efficiency, thermal stability, and physico-chemical properties, while their biological functionality was assessed using an *in vitro* approach with a rainbow trout (*Oncorhynchus mykiss*) liver-derived epithelial cell line (RTL-W1). Additionally, this study explored the potential of stabilizing a liposomal suspension through spray-drying into stable, dry formulations, representing a technological advantage in terms of improving handling, storage, and industrial scalability.

By combining encapsulation technology with *in vitro* screening, this study offers novel insights into the potential of AX as a functional feed ingredient, supporting the development of more stable, bioavailable, and economic viable AX formulations.

## 2. Results and Discussion

### 2.1. Encapsulation Assessment

Herein, three AX sources were employed: a natural source, namely a lipid extract from shrimp by-products (pigment 1), and two commercial synthetic alternatives (pigment 2 and 3). Shrimp by-products offer a promising avenue for sustainable AX extraction, supporting circular economy principles and reducing reliance on traditional extraction methods. The synthetic sources were chosen for their common use in feed industries due to cost-effectiveness and availability.

Among the variety of encapsulation techniques, spray-drying and liposome entrapment were selected for their complementary advantages in protecting AX and optimizing its use in aquafeeds. Spray-drying was chosen for its cost effectiveness, scalability, and ability to produce stable formulations suitable for large-scale feed production [25]. Liposome entrapment, in turn, enhances AX bioavailability, facilitates cellular uptake, and protects against oxidative stress [26]. Additionally, to improve stability and handling, liposomal formulations were further processed through spray-drying, enabling a comparative evaluation of encapsulation strategies that balance feasibility with functional performance in aquafeed applications.

#### 2.1.1. Encapsulation Efficiency

The encapsulation efficiency (EE) of the three encapsulated AX formulations showed no statistical difference between spray-drying and liposome entrapment (Figure S1). The EE of pigment 1 was 87% ± 0.01 and 89% ± 0.01, for pigment 2, it was 73% ± 0.03 and 78% ± 0.01, and for pigment 3, it was 85% ± 0.01 and 83% ± 0.04 for spray-drying and liposome entrapment, respectively. The obtained EE values align with previous studies on AX encapsulation. For instance, microcapsules containing AX from shrimp by-products, encapsulated by complex coacervation and spray-drying, showed an EE of 60% and 94%, respectively [19,31]. Furthermore, a study on liposomal encapsulation reported EE values around 90% for freeze-dried liposomes encapsulating various antioxidants extracted from natural waste [27].

The efficiency of encapsulation is closely linked to the chemical characteristics of AX, particularly its structural form and solubility [24]. Natural AX primarily occurs as mono- and diesters, where esterification with fatty acids increases its hydrophobicity and enhances oxidative stability. In contrast, synthetic AX is predominantly in its free form, which improves solubility but also renders it more vulnerable to oxidative degradation [7,32]. These differences can significantly impact the interaction between AX and the encapsulation matrix, affecting EE, pigment retention, and long-term stability in formulated feeds. High EEs can be generally achieved using materials that act as emulsifiers and film-forming agents, facilitating rapid crust formation in droplets and minimizing the diffusion of entrapped oil to the particle surface [33]. Additionally, the properties of wall and core materials, the emulsions, and the conditions of the drying process can all influence the efficiency and retention of core compounds. Herein, a relatively high EE of AX formulations was obtained with both techniques. The similar EE observed between spray-drying and liposome entrapment across the pigments might be attributed to the stabilization of liposomes through spray-drying, which standardized the final process, reducing variability [34]. Achieving comparable EE values between the two methods further demonstrates that encapsulation in liposomes was not only efficient but also adaptable for further processing into stable, dry formulations [27]. Based on the obtained results, both techniques effectively encapsulate various AX formulations, enhancing their stability regarding their original source.

#### 2.1.2. Particle Characterization

**Figure 1:**
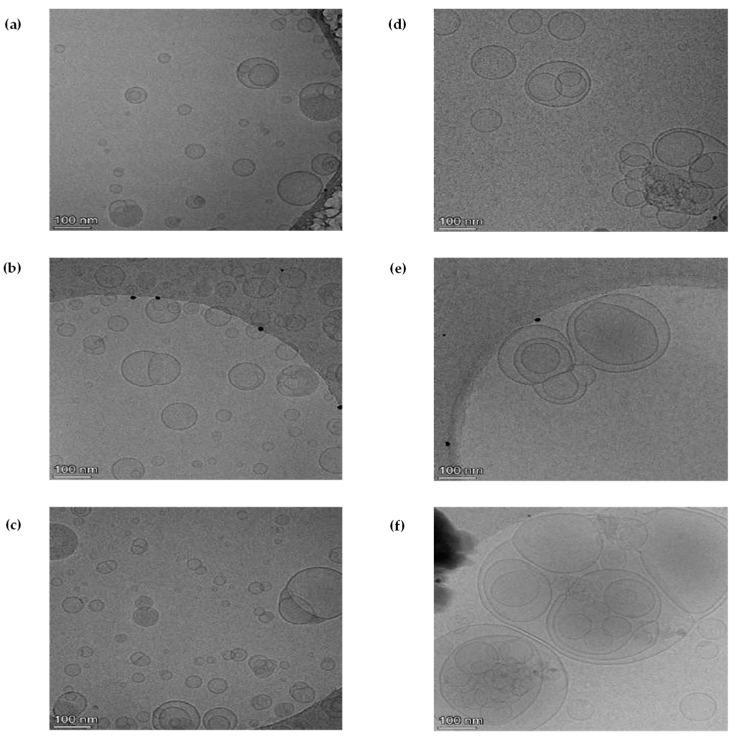
Cryo-transmission electron microscopy (cryo-TEM) images of the ultrastructure of fresh ((**a**–**c**), for pigment 1, 2, and 3, respectively) and rehydrated liposomes ((**d**–**f**), for pigment 1, 2, and 3, respectively) encapsulating astaxanthin (AX) from natural (pigment 1) and synthetic sources (pigment 2 and 3). Images were taken under low-dose conditions with a CETA-F camera.

The hydrodynamic properties of both fresh and rehydrated liposome-encapsulated AX were evaluated using Zetasizer Nano ZS (Table S1), while morphological characterization of liposomes was carried out through cryo-transmission electron microscopy (cryo-TEM) analysis (Figure 1). The hydrodynamic properties of liposomes revealed a significant increase in size between fresh (9.77 ± 0.04 nm for pigment 1; 11.67 ± 0.06 nm for pigment 2; 11.89 ± 0.03 for pigment 3) and rehydrated (768.64 ± 17.43 nm for pigment 1; 663.33 ± 37.86 nm for pigment 2; 607.27 ± 48.89 nm for pigment 3) liposome-encapsulated AX (Table S1). The polydispersity index (PDI), a measure of the particles’ size distribution (ranging from 0 to 1), indicated uniform distribution of the particles with values ranging from 0.23 ± 0.01 to 0.33 ± 0.03 for both fresh and rehydrated liposomes. Liposome stability also depends on the strength of interaction between droplets, which is determined by the charge on their surface. When this charge is sufficiently large, generally regarded as greater than ± 30 mV, the droplets are prevented from aggregating due to electrostatic repulsion between them [35,36]. The liposomal suspensions prepared in this study were therefore considered electrostatically stables as their zeta potential values ranged from −41.90 ± 0.20 to −55.63 ± 2.43 mV for both fresh and rehydrated liposomes.

Furthermore, the cryo-TEM analysis revealed that after the drying process, the liposomes underwent a rearrangement in their structure, with multiple liposomes coming together as bigger structures (Figure 1). The increase in size and rearrangement of the liposomes, after the drying process, is a well-known phenomenon resulting from the dehydration and rehydration processes [29,37,38]. During spray-drying, the removal of water can lead to structural rearrangements in the lipid bilayer, where liposomes may undergo a phase transition, leading to the formation of multilamellar structures and an increase in size upon rehydration [34]. Despite this variation, the PDI and zeta potential values of rehydrated liposomes remained unaffected. Maintaining electrostatic stability is crucial for encapsulated formulations, particularly for aquatic feed applications. Zeta potential measurements confirmed that liposomes maintained a strong negative charge, enhancing colloidal stability. These findings are particularly relevant for ensuring AX dispersion in water-based feed formulations, a common challenge in aquaculture.

**Figure 2:**
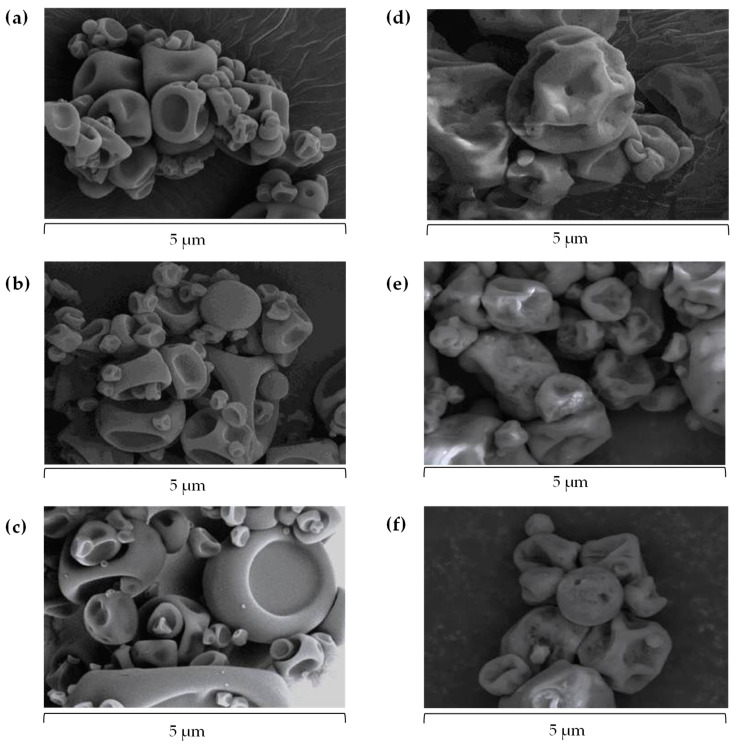
Scanning electron microscopy (SEM) images of the microstructure of astaxanthin (AX) microcapsules from natural (pigment 1) and synthetic (pigment 2 and 3) sources, encapsulated by spray-drying ((**a**–**c**), for pigment 1, 2, and 3, respectively) and liposome entrapment ((**d**–**f**), for pigment 1, 2, and 3, respectively). Images were captured at 5000× magnification.

After the drying process, the morphology of the particles was further investigated through scanning electron microscopy (SEM) analysis for all encapsulated AX formulations, highlighting the morphology of the wall materials used as encapsulating agents (maltodextrin and gum arabic, M-GA) (Figure 2).

Overall, the obtained images of the particles exhibited a rounded, partially collapsed morphology, a typical characteristic of these polymers processed by spray-drying, consistent with previous observations [19]. Particles directly spray-dried (Figure 2a–c) resulted in smoother surfaces compared to the liposomal encapsulation particles, which resulted in rougher particle surfaces (Figure 2d–f), likely due to their complex, bilayered structure. When directly spray-dried, the size distribution of the particles was broad: 4.82 ± 2.15 µm, 5.39 ± 2.18 µm, and 6.37 ± 2.08 µm for pigment 1, 2, and 3, respectively. When encapsulated in liposomes and stabilized into dry powders, the size distribution of the particles was 7.50 ± 2.57 µm, 6.83 ± 2.66 µm, and 7.09 ± 2.96 µm, for pigment 1, 2, and 3, respectively, with a significant increase in size observed for pigment 1 and 2 compared to their directly spray-dried counterpart. The increase in size when AX was encapsulated in liposomes was expected due to the additional structural components introduced during formation and further stabilization into dry forms [39]. The present findings align with a previous study, where microcapsules of AX encapsulated by spray-drying resulted in a particle size with a 6 µm diameter, round in shape, and partially collapsed, using M-GA as encapsulating agents [19]. Both techniques employed in this study led to a stable, powdered formulation, which is particularly noteworthy for liposomal stabilization by spray-drying as this represents a technological advantage in terms of improving the physical stability and half-life of AX, preventing degradation or oxidation during storage, and facilitating handling and transportation.

#### 2.1.3. Colour Measurement

The colorimetric parameters of the encapsulated AX formulations are reported in Table S2. The application of spray-drying and liposome entrapment revealed distinct effects on the characteristics of the pigments. AX encapsulated in liposomes, further stabilized by spray-drying, significantly increased the hue (h°), compared to directly spray-dried AX, suggesting a shift in colour perception toward a lighter or more yellowish tone. Liposome entrapment also significantly influenced the chroma (C*) values differently, enhancing the saturation and intensity of synthetic pigments while diminishing these qualities in the natural pigment, resulting in a less saturated colour. The stabilization of liposomes likely contributed to these changes by altering the pigment’s interaction with light, possibly due to interactions with the lipid bilayer [37]. Lightness (L*) also significantly increased in the natural pigment, indicating brighter appearance, but decreased in the synthetic pigments, producing a darker colour compared to directly spray-dried counterparts. Colour is a key factor influencing consumer perception and market acceptance of aquaculture products, where pigmentation is often associated with quality and nutritional value [40]. Herein, higher chroma in synthetic AX formulations could enhance pigment deposition efficiency, leading to a more intense and visually appealing coloration, possibly increasing consumer preference and commercial value. Conversely, the reduction in chroma observed in natural AX with liposomal encapsulation may affect its ability to achieve the desired pigmentation, potentially limiting its competitiveness in the global market. Additionally, the observed hue shifts could alter the final coloration of the flesh, which may need to align with species-specific and consumer expectations. This underscores the importance of encapsulation method selection, as the approach not only influences the potential functionality and stability of the core compounds but also affects the perceived optical properties of the final product. Further research is needed to assess pigment deposition efficiency in fish fillets, particularly in comparison with non-encapsulated AX formulations.

#### 2.1.4. Hygroscopicity

No statistical difference in hygroscopicity between spray-drying and liposome entrapment was observed across all encapsulated AX formulations (Figure S2). The hygroscopicity values, expressed as grams (g) of water absorbed in 100 g of powder, were 19.68 ± 0.55 and 21.67 ± 2.89 for pigment 1, 25.79 ± 2.48 and 26.67 ± 5.77 for pigment 2, and 22.02 ± 2.64 and 24.26 ± 3.94 for pigment 3 for AX formulations encapsulated by spray-drying and liposome entrapment, respectively. Values ranging from 5 to 20 g of water absorbed per 100 g of powder are reported in previous studies while working with a gelatine–gum Arabic complex [41,42]. The data obtained in the present study are on the upper end of this range, suggesting that while both encapsulation techniques employed were effective, the composition and nature of AX, as well as the choice of specific encapsulating agents, may overall influence moisture absorption characteristics. Using a mixture of M-GA as encapsulating agents, likely due to the presence of polar groups in these polymers which may attract and hold water molecules, resulted in wall materials with good solubility and rapid reconstitution in water [19]. Nevertheless, the relatively higher degree of water absorption observed in all encapsulated AX formulations also suggests that both encapsulation methods do not mitigate water absorption over time.

Hygroscopicity, a material’s ability to absorb moisture from its surroundings, significantly influences product quality and performance [43]. Measuring this parameter is important during encapsulation studies, particularly for products like AX, where stability, efficiency, and shelf-life are crucial. Additional research might be directed toward understanding and controlling this parameter during the encapsulation of bioactive compounds in order to improve storage conditions, prolong shelf-life, and preserve efficacy for a longer period of time.

#### 2.1.5. Temperature Stability

**Figure 3:**
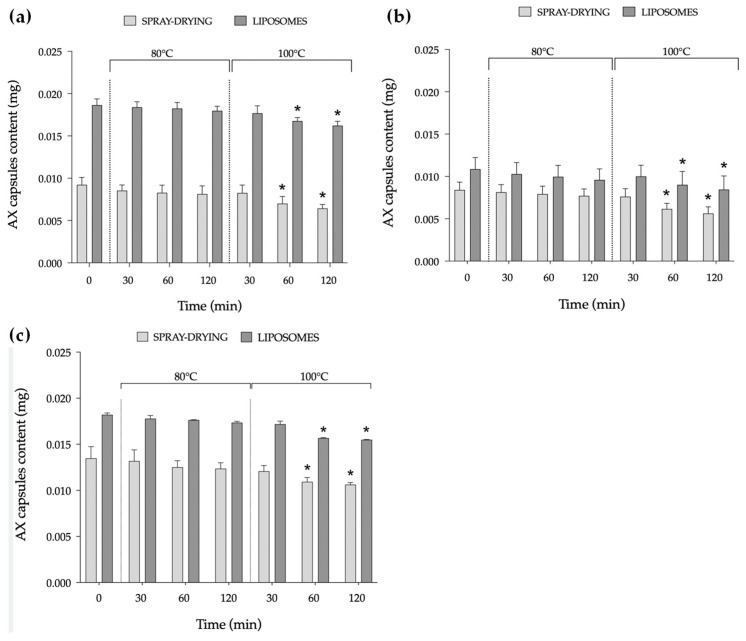
The temperature stability of encapsulated astaxanthin (AX) formulations from natural (pigment 1) and synthetic sources (pigment 2 and 3), encapsulated by spray-drying (dark grey) and liposome entrapment (light grey). Pigment 1 (**a**), pigment 2 (**b**), and pigment 3 (**c**) were exposed to two temperatures (80° and 100 °C) for 30, 60, and 120 min. Time 0 represents the original AX content of the capsules measured with the encapsulation efficiency (EE) after the encapsulation process. The results are reported as the mean ± standard deviation and expressed as mg of AX content of the capsules (*n* = 3). Asterisks (*) denote significant differences (*p* < 0.05) compared to time 0.

To assess the thermal stability of the encapsulated AX formulations under representative conditions for fish feed production, each AX formulation was subjected to subsequent thermal treatments at 80 °C and 100 °C for 30, 60, and 120 min (Figure 3). At 80 °C, all encapsulated AX formulations exhibited good stability, without significant differences compared to the original AX content of the capsules. Under the selected temperature, both techniques effectively protected AX from thermal degradation. This level of stability is particularly advantageous for incorporation into fish feed, where moderate heat resistance is required during the extrusion process [44]. However, at 100 °C, a significant decrease in AX content was observed starting after 60 min of exposure and varied with the encapsulation method.

Notably, at 30 min, the AX content remained stable across pigments and between the two techniques. This stability is particularly noteworthy considering the gradual temperature increase in the extrusion process, where the material is not exposed to high temperatures for prolonged periods.

The stability of AX, like other carotenoids, is known to strongly depend on external conditions (such as temperature, light, oxygen, pH changes) [45], and only few studies have comprehensively investigated these aspects. Regarding temperature, a previous study assessed the effect of moderate and high temperatures (60, 90, and 120 °C) on the stability of AX using sodium caseinate (SC) and modified lecithin (ML) as encapsulating agents [30]. The decrease in retention of AX as the temperature increased was significant in both SC- and ML-stabilized nanoemulsions, with a significant increase in droplet size observed at the highest tested temperature. Other studies conducted at lower temperatures (25, 35, and 45 °C) aimed to assess the retained pigment quality of AX microcapsules [46]. Although some fluctuations were observed, none of the treatments showed significant decreases in AX content. Conversely, significant differences in the half-life and antioxidant activity of AX microcapsules at 30, 40, and 50 °C have been observed using different encapsulating agents, including M-GA [47].

Heat stability is a fundamental consideration in the application of AX in aquafeeds, particularly during extrusion and pelletization processes. These manufacturing techniques involve thermal processing, which can influence the retention and functionality of AX. To ensure its effectiveness, it is crucial to minimize potential losses by optimizing formulation strategies, such as encapsulation, and adjusting processing conditions to maintain the integrity of AX throughout feed production. In the present study, both techniques effectively prevented AX degradation at 80 °C, regardless of its natural or synthetic source. While these preliminary results highlight the potential stability of AX under initial processing conditions, further research is needed to evaluate its behaviour in real feed formulations throughout the full feed processing phase.

### 2.2. In Vitro Study

#### 2.2.1. Cell Viability Assay

**Figure 4:**
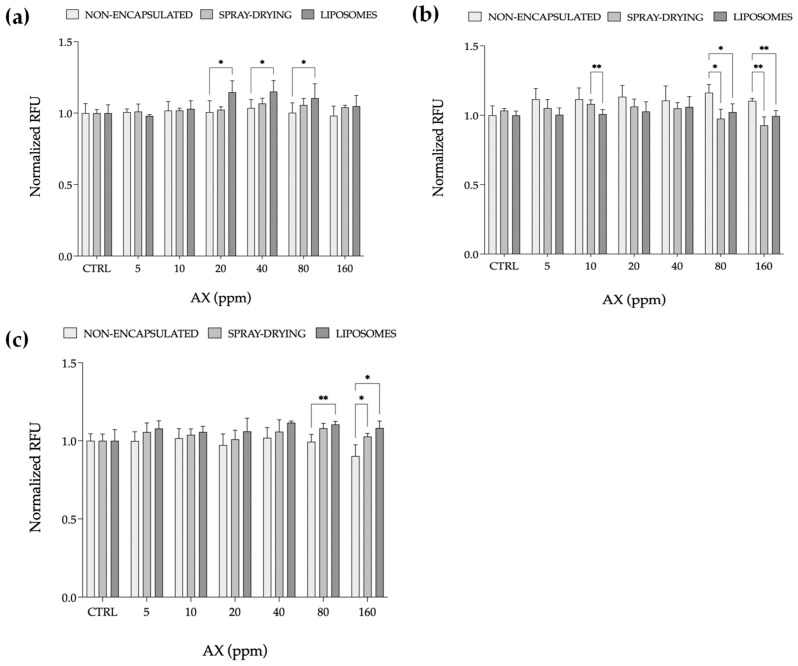
Cell viability of RTL-W1, a liver-derived cell line from rainbow trout (*Oncorhynchus mykiss*), exposed for 24 h to AX from natural (pigment 1) (**a**) and synthetic (pigment 2 and 3) (**b**,**c**) sources before and after the encapsulation processes. The results are reported as relative fluorescence units (RFUs), normalized to the control, and expressed as the mean ± standard deviation (*n* = 5). Asterisks denote statistical differences (* *p* < 0.05; ** *p* < 0.01) between encapsulation techniques, including the non-encapsulated form for each tested concentration.

**Figure 5:**
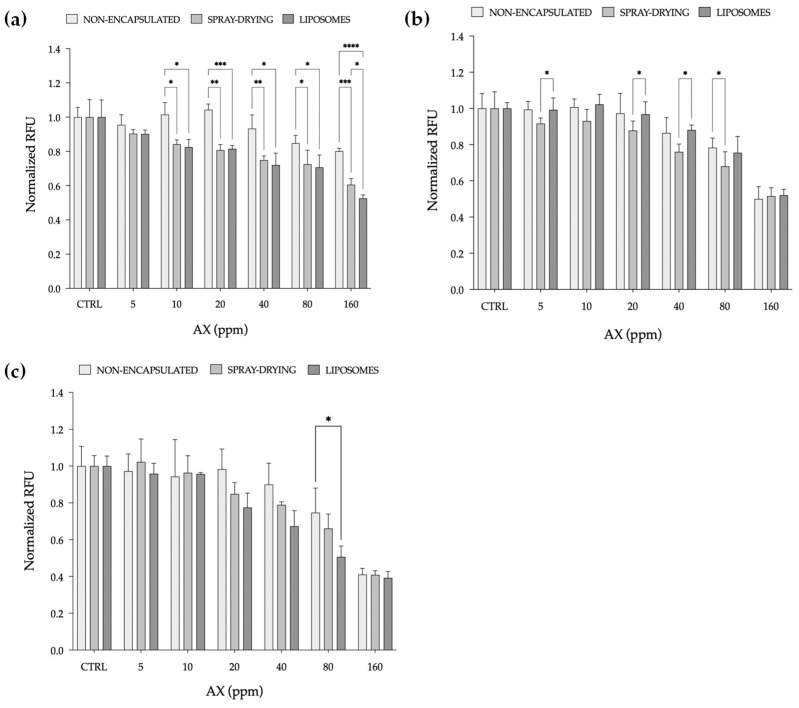
Reactive oxygen species (ROS) production of RTL-W1, a liver-derived cell line from rainbow trout (*Oncorhynchus mykiss*), exposed for 1 h to astaxanthin (AX) from natural (pigment 1) (**a**) and synthetic (pigment 2 and 3) (**b**,**c**) sources before and after the encapsulation processes. The results are reported as relative fluorescence units (RFUs), normalized to the control, and expressed as the mean ± standard deviation (*n* = 5). Asterisks denote statistical differences (* *p* < 0.05; ** *p* < 0.01; *** *p* < 0.001; **** *p* < 0.0001), between encapsulation techniques, including the non-encapsulated form for each tested concentration.

The results of cell viability obtained by exposing RTL-W1 cells to each AX pigment, both encapsulated and non-encapsulated, are presented in Figure 4. Encapsulation in liposomes generally promoted cell viability more effectively than directly spray-dried forms (Figure 5a,c), with the exception of pigment 2, which appeared to significantly decrease at the highest tested concentrations (80 and 160 ppm) compared to the non-encapsulated form, and at 10 ppm between the two techniques (Figure 4b). The reduction in viability observed was not indicative of cytotoxicity effects of AX, considering that the encapsulated pigment still maintained good viability levels similar to the control group. This could suggest that the decrease reflected moderation of cellular activity compared to the non-encapsulated form rather than cytotoxic effects.

Overall, the obtained results align with a previous study, showing that no cytotoxic effects of AX were observed in four fish cell lines (SAF-1, DLB-1, FuB1, and PLHC-1) [48]. Indeed, AX increased the viability of two of them, DLB-1 and PLHC-1. In the present study, for RTL-W1, which served as a model for fish liver function, cell viability, typically measured with assays that detect cellular metabolic activities, is a valuable method that reflects how liver cells respond to bioactive compounds like AX [49]. Cell viability was assessed through a widely used metabolic indicator (AlamarBlue) that relies on the ability of viable cells to reduce resazurin to resorufin, producing a quantifiable fluorescence signal proportional to cellular metabolic activity [50]. Its compatibility with encapsulated and non-encapsulated AX offers further insights into how delivery systems influence bioavailability and cellular responses, making it ideal for studying the impact of functional feed ingredients like AX in fish cell models.

#### 2.2.2. ROS Production Assay

The inherent ROS production of RTL-W1 cells exposed to each AX formulation, both encapsulated and non-encapsulated, is presented in Figure 5. A significant decrease in ROS production starting from 10 ppm was observed after the encapsulation of pigment 1 (Figure 5a), regardless of the technique applied, compared to the non-encapsulated form, which did not display any significant decrease in ROS while increasing AX concentration. A significant decrease in ROS compared to the non-encapsulated form was also observed at 80 ppm (Figure 5b) when pigment 2 was directly spray-dried. When comparing the two methods, the results show a significant decrease in ROS production at 5, 20, and 40 ppm. Finally, with pigment 3 (Figure 5c), after the encapsulation, the production of ROS slightly decreased while increasing the concentration of AX, with a significant decrease observed only at 80 ppm compared to the non-encapsulated counterpart.

The observed decrease in ROS production further strengthens the evidence of encapsulation techniques influencing bioavailability and cellular responses and enhancing the effectiveness of AX compared to their non-encapsulated counterparts. Overall, the obtained data show that the antioxidant power of AX increased as the concentration increased, which is consistent with the expected behaviour of antioxidants [51,52].

In recent years, there has been a growing interest in AX not only for flesh pigmentation but also due to its strong antioxidant properties, particularly within the context of fish physiology [53,54]. The higher antioxidant power of AX is largely attributed to its molecular structure with the presence of hydroxyl (OH) and keto-moieties (C=O) on each ionone ring [55,56,57]. The powerful antioxidant capacity of AX is correlated with its strong electron-donating capability to neutralize endogenously produced free radicals and converting them into more stable products [58,59,60], thereby reducing their reactivity and mitigating the detrimental impact of oxidative stress [61,62].

AX’s role in modulating oxidative stress in fish offers promising benefits in aquaculture, representing a potential strategy to enhance fish resilience [60]. Through its capacity to augment antioxidant enzyme activities, AX supplementation may offer a means to mitigate adverse effects and promote fish well-being and performance [61,62]. Encapsulation further amplifies these effects, improving the stability and bioavailability of AX, thereby enhancing its antioxidant power, as documented in the present study.

By combining encapsulation technology with an *in vitro* screening approach, this study provides novel insights into the potential of AX as a functional feed ingredient. Unlike previous studies that typically focused on a single technique or compare multiple techniques without assessing their impact on physico-chemical stability and biological functionality in aquaculture applications, our research uniquely combines these aspects to assess how encapsulated AX formulations influence cellular responses at the molecular level. This approach not only highlights the role of encapsulation in enhancing AX stability and bioavailability but also further strengthens the value of fish cell lines as a rapid and ethical tool for evaluating functional feed ingredients.

## 3. Materials and Methods

### 3.1. Astaxanthin Sources

For this study, both natural and synthetic sources of AX were selected: one natural source derived from shrimp by-products as a lipid extract, and two commercially synthetic alternatives (Table S3). Within this work, the natural source is referred to as pigment 1, while the synthetic sources are referred to as pigment 2 and 3.

This study did not seek to directly compare the different AX sources; rather, it focuses on investigating the encapsulation techniques applied to different AX sources to enhance their effectiveness as functional ingredients, promoting better incorporation into fish feeds.

### 3.2. Shrimp Lipid Extraction

Frozen shrimps (*Litopenaeus vannamei*) were thawed at room temperature and peeled manually. Cephalothorax, cuticles, pleopods, and tails were homogenized using a domestic blender. Aliquots of 100 g of homogenate were mixed with 500 mL of ethyl acetate and stirred at room temperature for 30 min in the dark. The solution was then filtered using Whatman No. 1 filter paper (Whatman International Ltd., Maidstone, UK), and the filtrate was then evaporated using a rotary evaporator (Büchi R-300, Büchi Labortechnik AG, Flawil, Switzerland) to remove the solvent from the sample. The final extract was stored at −20 °C until use.

### 3.3. Encapsulation Processes

#### 3.3.1. Spray-Drying

A mixture of maltodextrin and gum Arabic (M-GA) (Manuel Riesgo S.A., Madrid, Spain) in a 50:50 ratio (*w*/*w*) was dissolved in distilled water at 20 g/100 mL [19]. Then, 70 mg of AX was added to a 500 mL of M-GA solution (70 mg AX/100 g M-GA). The emulsion was obtained by homogenization in Ultra-Turrax T25 (IKA-Werke GmbH & Co., Staufen, Germany) at 14,000 rpm for 1 min and subsequently spray-dried using a B-290 spray-dryer (Büchi R-300, Büchi Labortechnik AG, Flawil, Switzerland), with the following processing conditions: inlet temperature of 150 °C; outlet temperature of 88 ± 2 °C; aspiration rate of 90%; flow rate of 6 mL/min.

#### 3.3.2. Liposome Entrapment

Fresh liposomes were obtained following the protocol described by Marín et al. [27], but slightly modified. Briefly, 1.25 g of AX was dissolved in 500 mL of 0.2 M phosphate buffer (pH 7), following the addition of 25 g of rapeseed lecithin (5 g AX/100 g lecithin) (BungeMaxx, Amsterdam, The Netherlands), with gentle agitation. The solution was then placed in a water bath for 1 h at 80 °C, followed by sonication in Q-Sonica (Q700 model, Qsonica sonicators, Newton, MA, USA), set at a 90% amplitude, during five consecutive cycles of 1 min each with 1 min stop. Subsequently, the obtained liposomal solutions were further stabilized by spray-drying in powdered forms, using the same mixture of polymers (M-GA, 50:50 ratio *w*/*w*) previously described. The processing conditions were as follows: inlet temperature of 150 °C; outlet temperature of 92 ± 1 °C; aspiration rate of 80%; flow rate of 6 mL/min.

### 3.4. Encapsulation Properties

#### 3.4.1. Encapsulation Efficiency

The encapsulation efficiency (EE) was calculated by quantifying the total amount of AX inside the capsules in comparison with the amount of AX on their surface, expressed as percentage. To quantify the amount of surface AX, 400 mg of powder was weighed (*n* = 3) and washed twice with 5 mL of hexane. The two volumes of hexane where then mixed, and the surface AX content was calculated as defined by Britton et al. [63] according to Equation (1):

(1) $$\mathrm{Carotenoid}\;(\mathrm{mg})=\frac{A\times V\times P}{ɛ}$$

where A is the absorbance at 468 nm, V is the dilution volume (mL), P is the molecular weight, and *ε* is the molar absorption coefficient of AX (L mol^−^^1^ cm^−^^1^) (597 and 125,000, respectively).

To determine the total amount of AX encapsulated, 50 mg of powder was weighed (*n* = 3) and dissolved in 2 mL of distilled water. Then, 3 mL of hexane was added and vortexed, followed by centrifugation at 5000× *g* for 15 min at room temperature. The upper hexane phase was collected, and the process was repeated twice in the same conditions until the hexane phase was colourless. The hexane phases were mixed, and the total AX content was measured by absorbance and calculated using Equation (1). The EE was calculated according to Equation (2):

(2) $$\mathrm{EE}\;(\%)=\frac{\left(total\;AX-surface\;AX\right)}{total\;AX}\times 100$$

#### 3.4.2. Microstructure and Ultrastructure Analysis

After the drying process, the microstructure of each encapsulated AX formulation was determined by scanning electron microscopy (SEM) analysis, using a JSM-IT700HR (JEOL, Tokyo, Japan) electron microscope. The samples were placed in an aluminium sample holder, kept at 0% RH for 7 days, and treated with gold–palladium immediately prior to analysis. Images were captured at 5000× and 10,000× magnification [19].

The ultrastructure of both fresh and rehydrated liposome-encapsulated AX (125 mg AX/2 mL distilled water) was evaluated by cryo-transmission electron microscopy (cryo-TEM) analysis. Samples were applied to holey carbon grids (Quantifoil) after glow-discharge and immediately blotted and vitrified using a FEI Vitrobot cryo-plunger. Micrographs were taken at 72,000× magnification in a TALOS L120C electron microscope, operated at 120 kV and equipped with a Gatan liquid nitrogen specimen holder for cryo-TEM. Images were taken under low-dose conditions with a CETA-F camera (ThermoFisher Scientific, Waltham, MA, USA).

#### 3.4.3. Hydrodynamic Properties and Particle Size Measurement

The hydrodynamic properties of both fresh and rehydrated liposome-encapsulated AX were measured in triplicate (*n* = 3) using Zetasizer Nano ZS (Malvern Instruments Ltd., UK) [64]. Hydrodynamic size was expressed in nm, zeta potential in mV, and the polydispersity index (PDI) is dimensionless. The particle size of spray-dried microcapsules containing AX was measured by image analysis over the SEM pictures using Image J software (Version 1.53t, Wayne Rasband, National Institute of Health, USA). The diameter of 50 particles was measured, and the results were expressed in µm as the mean ± standard deviation.

#### 3.4.4. Colour Measurement

The colorimetric parameters of the encapsulated AX formulations were measured using a Konica Minolta CM-3500d spectrophotometer (Konica, Minolta Sensing Inc., Osaka, Japan) set to D65 illuminant/10° observer. The CIELAB color space was used to obtain the colour coordinates L*, a*, and b*, which were then converted to calculate C* and h°. The results were expressed as L*h°C* colour coordinates, where L* is the luminosity, which refers to the brightness of the colour, ranging from 0 (black) to 100 (white); h° is the hue, which refers to the attribute of colour perceived, typically measured in degrees on the colour wheel (0° to 360°); and C* is the chroma, which refers to the vividness or intensity of the colour [65].

#### 3.4.5. Hygroscopicity

To determine the hygroscopicity of the encapsulated AX formulations, 400 mg of powder (*n* = 3) was placed in a desiccator containing a saturated solution of sodium sulphate (Na_2_SO_4_) at 25 °C (RH~80%) for a week [43]. The hygroscopicity was determined by measuring the mass of water absorbed per 100 g of powder after 7 days.

#### 3.4.6. Temperature Stability

A temperature stability study was carried out to assess the stability of the encapsulated AX formulations under relevant temperatures for the incorporation of AX into fish feed through an extrusion process. To do so, 400 mg of powder was weighed (*n* = 3) and subjected to two temperatures (80 and 100 °C) for 30, 60, and 120 min. AX release from the wall materials was calculated by quantifying the surface AX content, using Equation (1) as previously described (Section 3.4.1). Then, the AX content of the capsules was calculated by using Equation (3):

AX content (mg): total AX − surface AX(3)

We used the total amount of AX encapsulated, previously measured, to determine the EE of the pigments.

### 3.5. In Vitro Study

#### 3.5.1. RTL-W1 Cell Culture

Rainbow trout (*Oncorhynchus mykiss*) epithelial liver cells, RTL-W1, kindly provided by the Fraunhofer Institute for Molecular Biology and Applied Ecology (Fraunhofer IME, Schmallenberg, Germany), were cultured in 75 cm^2^ tissue culture flasks (Corning Inc., Corning, NY, USA), supplemented with 10% fatal bovine serum (Biowest, Nuaillé, France), 100 U/mL penicillin, and 100 µg/mL streptomycin (Gibco, Waltham, MA, USA). Confluent cells were seeded in 96-well plates, with a density of 3 × 10^4^ cells per well, and exposed to each AX pigment (both encapsulated and non-encapsulated forms) in a range of concentrations from 5 to 160 ppm. Exposure solutions were freshly prepared by dilution of AX in L-15 media to perform the cell viability assay, and phosphate-buffered saline (PBS) (VWR, International LLC, Radnor, PA, USA) was used to assess reactive oxygen species (ROS) production. Untreated cells in L-15/PBS were regarded as a control, L-15/PBS alone (w/o cells) was regarded as a blank, and cells exposed to hydrogen peroxide (H_2_O_2_, 100 µM) were regarded as a positive control.

#### 3.5.2. Cell Viability Assay

Cell viability was assessed using the fluorometric method described by Kamiloglu et al. [50]. Briefly, confluent cells were exposed for 24 h to each AX pigment. Then, the exposure solution was removed and resazurin dye (AlamarBlue TM, ThermoFisher Scientific, Waltham, MA, USA) was added in a 1:10 ratio with L-15. The fluorescence intensity of viable cells was quantified using a microplate reader (SpectraMax Paradigm Multi-Mode, Molecular Devices LLC, San Jose, CA, USA) at excitation and emission wavelengths of 530 and 590 nm, respectively. Relative fluorescence units (RFUs) were normalized to the control. For each concentration five replicates were tested (*n* = 5).

#### 3.5.3. ROS Production Assay

The production of ROS was measured by applying a modified version of the protocol described by LeBel et al. [66]. The 2′,7′-dichlorodihydrofluorescin diacetate (H2DCF-DA) (ThermoFisher Scientific, Waltham, MA, USA) probe was used as an ROS production indicator and 100 µL of 10 mM solution was added to each well (except the blank) for an incubation time of 30 min. After removing the probe, RTL-W1 cells were exposed for 60 min to each AX pigment. The fluorescence emitted due to the oxidation of H2DCF-DA was read using a microplate reader (SpectraMax Paradigm Multi-Mode, Molecular Devices LLC, San Jose, CA, USA) at excitation and emission wavelengths of 485 nm and 528 nm, respectively. RFUs were normalized to the control. For each concentration, five replicates were tested (*n* = 5).

### 3.6. Statistical Analysis

GraphPad prism 9 (GraphPad software LLC, San Diego, CA, USA) was used to perform the statistical analysis of the data. Normality distribution of the data was assessed with the Shapiro–Wilk test. Normally distributed data were analysed using a t test and two-way ANOVA. In addition, for the two-way ANOVA, the Geisser–Greenhouse correction was also applied when the hypothesis of sphericity was violated. A post hoc test (Tuckey’s multiple comparison test) was also performed to identify which groups differ from each other. Letters and asterisks were used to denote significant differences (*p* < 0.05). When the data were not normally distributed, Mann–Whitney and Kruskal–Wallis tests were used.

According to the purpose of this study, only the comparison carried out between the two encapsulation techniques is discussed within the manuscript.

## 4. Conclusions

The present study explored the potential of encapsulation techniques to enhance the effectiveness of AX as a functional ingredient, addressing key challenges related to its low bioavailability and susceptibility to degradation and oxidation in its non-encapsulated form. Both techniques offer clear advantages in terms of AX stabilization and bioavailability, yet economic feasibility and scalability remain critical factors for large-scale applications. Spray-drying, being an established industrial technique, provides a cost-effective and scalable solution for encapsulating AX, allowing for continuous production and easy integration into current aquafeed processing lines. Its low production cost and high processing capacity make it the most practical option for large-scale applications. Nevertheless, spray-dried AX formulations may offer lower bioavailability compared to liposome entrapment, as the encapsulation matrix does not actively enhance AX absorption in fish tissues. Liposomal encapsulation, by contrast, significantly improves AX bioavailability due to its phospholipid bilayer structure, which facilitates cellular uptake. Despite these advantages, liposome production is not yet optimized for large-scale feed manufacturing due to its batch-wise nature and reliance on high-purity phospholipids, which increases production costs. Furthermore, liposomal formulations require additional stabilization to enhance storage stability, adding to processing complexity. A key finding of this study is that stabilizing liposomal AX via spray-drying could serve as a feasible approach, combining the enhanced bioavailability of liposomes with the cost-effectiveness of spray-drying. While this offers a promising compromise, further research is needed to optimize production efficiency, reduce costs, and evaluate the feasibility of integrating this approach into commercial aquafeeds.

Additionally, this study supports the utilization of alternative natural sources of AX, such as shrimp by-products, reinforcing the principles of a circular economy and sustainability in aquaculture. By valorising seafood processing waste, this approach aligns with the broader goal of enhancing resource efficiency and reducing environmental impact in fish feed production.

## Data Availability

The original data presented in the study are reported in an excel file in the Supplementary Materials. Further inquiries can be directed to the corresponding author.

## Supplementary Materials

The following supporting information can be downloaded at https://www.mdpi.com/article/10.3390/md23040143/s1: Figure S1. Encapsulation efficiency; Table S1. Zeta potential measurement of liposomes; Table S2. Colour measurement; Figure S2. Hygroscopicity; Table S3. Astaxanthin sources.

## Abbreviations

The following abbreviations are used in this manuscript:

AX	Astaxanthin
EE	Encapsulation efficiency
H2DCF-DA	2′,7′-dichlorodihydrofluorescin diacetate
M-GA	Maltodextrin–gum Arabic
ML	Modified lecithin
PBS	Phosphate-buffered saline
PDI	Polydispersity index
PUFAs	Polyunsaturated fatty acids
RFU	Relative fluorescence units
ROS	Reactive oxygen species
SC	Sodium caseinate
SEM	Scanning electron microscopy
TEM	Transmission electron microscopy
