# Supplementary material for: Encapsulation Techniques to Enhance Astaxanthin Utilization as Functional Feed Ingredient
Source: Mar Drugs. 2025 Mar 26;23(4):143. doi: 10.3390/md23040143 (PMC12028729; doi:10.3390/md23040143)
Supplement: Supplementary file 1 [file marinedrugs-23-00143-s001.zip › Supplementary Materials.pdf]

SUPPLEMENTARY MATERIALS

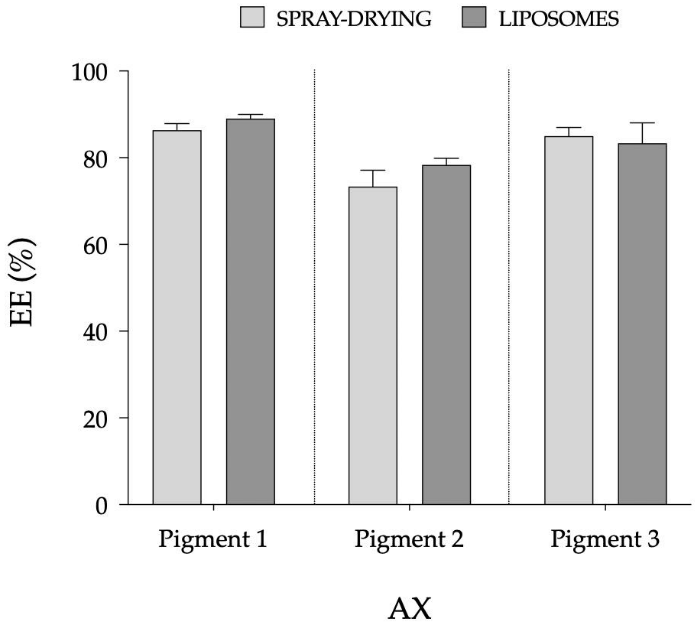

Figure S1. Encapsulation efficiency (EE) of astaxanthin (AX) from natural (pigment 1) and synthetic (pigment 2 and 3) sources, encapsulated by spray-drying (light grey) and liposome entrapment (dark grey), respectively. Results are reported as percentage (%) of EE and expressed as mean ± standard deviation (n =3).

Table S1. Hydrodynamic size (nm), polydispersity index (PDI) and zeta (ζ) potential (mV) of fresh and rehydrated liposomes-encapsulating AX from natural (pigment 1) and synthetic (pigment 2 and 3) sources. The results are reported as mean ± standard deviation (n = 3). Asterisks (*) denote significant differences ($p < 0.05$) between fresh and rehydrated liposomes-encapsulating each AX pigment.

LIPOSOMES	SIZE (nm)	PDI	ζ – POTENTIAL (mV)
Pigment 1			
Fresh	9.77 ± 0.04*	0.27 ± 0.01	-52.57 ± 4.65
Rehydrated	768.64 ± 17.43	0.32 ± 0.06	-55.63 ± 2.43
Pigment 2			
Fresh	11.67 ± 0.06*	0.23 ± 0.01	-50.40 ± 4.93
Rehydrated	663.33 ± 37.86	0.24 ± 0.06	-43.53 ± 1.31
Pigment 3			
Fresh	11.89 ± 0.03*	0.27 ± 0.01	-51.93 ± 7.19
Rehydrated	607.27 ± 48.89	0.33 ± 0.03	-41.90 ± 0.20

Table S2. Color parameters: lightness (L*), hue (h°), and chroma (C*) of the encapsulated AX from natural (pigment 1) and synthetic (pigment 2 and 3) sources, encapsulated by spray-drying and liposome entrapment. Results are reported as mean ± standard deviation (n = 3). Asterisks (*) denote significant differences ($p < 0.05$) between the two techniques for each color parameter.

ENCAPSULATION	L*	h°	C*
Pigment 1			
Spray-drying	76.97 ± 0.57*	48.87 ± 0.02*	32.19 ± 0.28
Liposomes	85.43 ± 0.42	72.54 ± 0.16	17.68 ± 0.20*
Pigment 2			
Spray-drying	89.16 ± 0.16	41.46 ± 0.10*	12.01 ± 0.08*
Liposomes	69.30 ± 0.09*	47.80 ± 0.12	35.56 ± 0.72
Pigment 3			
Spray-drying	88.78 ± 0.32	40.05 ± 0.28*	12.11 ± 0.16*
Liposomes	72.13 ± 0.08*	50.72 ± 0.12	38.31 ± 0.36

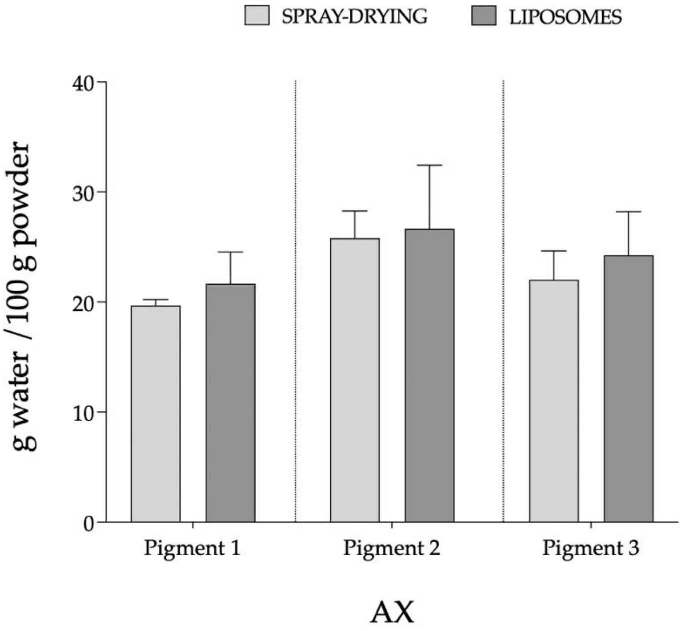

Figure S2. Hygroscopicity of encapsulated AX from natural (pigment 1) and synthetic (pigment 2 and 3) sources, encapsulated by spray-drying (light grey) and liposome entrapment (dark grey). Results are reported as mean ± standard deviation, and expressed as g of water absorbed in 100 g of powder, after 7 days of storage (n = 3).

Table S3. Summary of the astaxanthin (AX) sources used in this study, including their supplier, AX content, and nomenclature used within the manuscript.

AX	SOURCE	SUPPLIER	AX CONTENT
**Pigment 1**	Natural	Shrimps (*L. vannamei*) (Angulas Aguinaga Burgos, Spain)	~ 1%
**Pigment 2**	Synthetic	DIVIS (DIVIS Nutraceuticals, Germany)	~ 10%
**Pigment 3**	Synthetic	DSM (DSM Nutritional Products, Switzerland)	~ 10%
